# Supplementary material for: Targeted metabolomics investigation of metabolic markers of Mycobacterium tuberculosis in the cerebrospinal fluid of paediatric patients with tuberculous meningitis
Source: PLoS One. 2024 Dec 17;19(12):e0314854. doi: 10.1371/journal.pone.0314854 (PMC11651539; doi:10.1371/journal.pone.0314854)
Supplement: S1 File — (DOCX) [file pone.0314854.s002.docx]

**SUPPLEMENTARY INFORMATION:**

**Targeted metabolomics investigation of metabolic markers of *Mycobacterium tuberculosis* in the cerebrospinal fluid of paediatric patients with tuberculous meningitis**

**Victory Samuel and Shayne Mason***

**Biochemistry Department, Focus Area for Human Metabolomics, North-West University, Potchefstroom, South Africa**


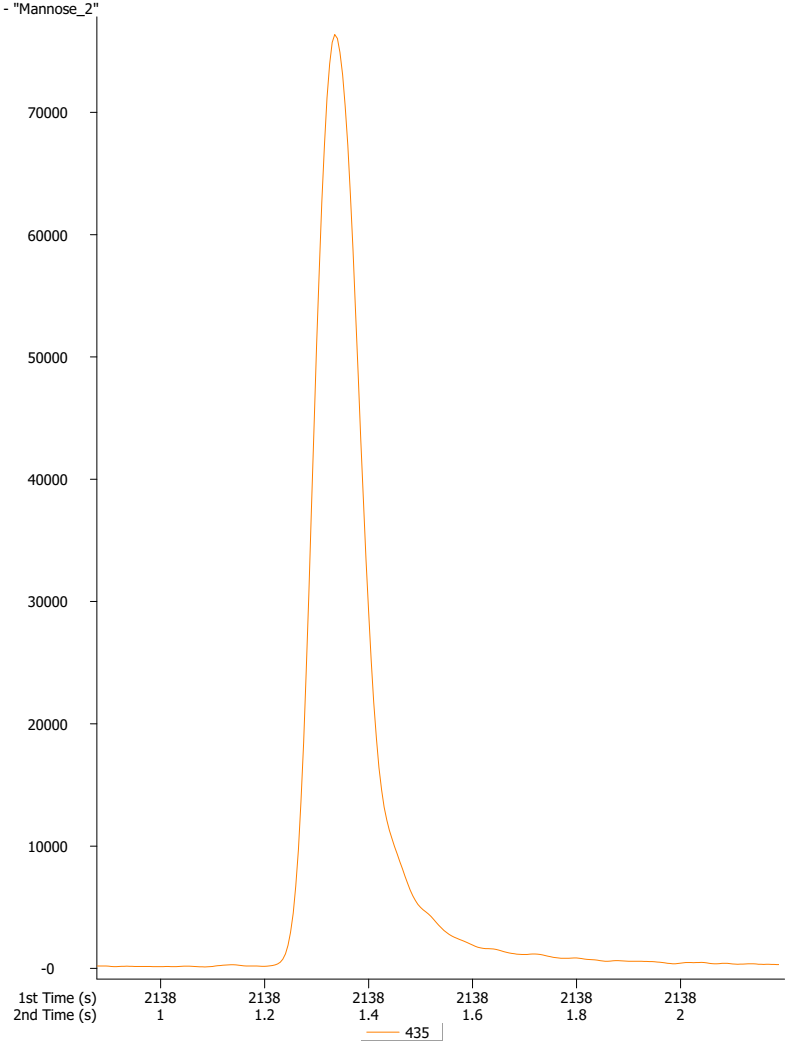

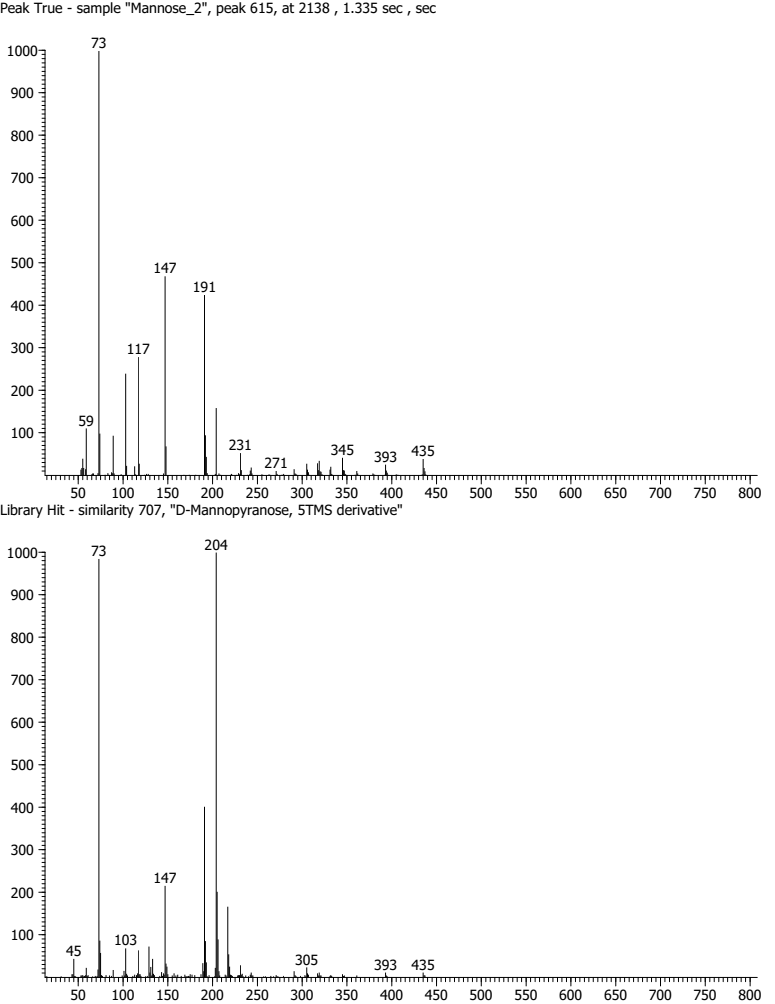


**Figure S1: Gas chromatogram (left) and MS pattern (right) of D-mannose.**


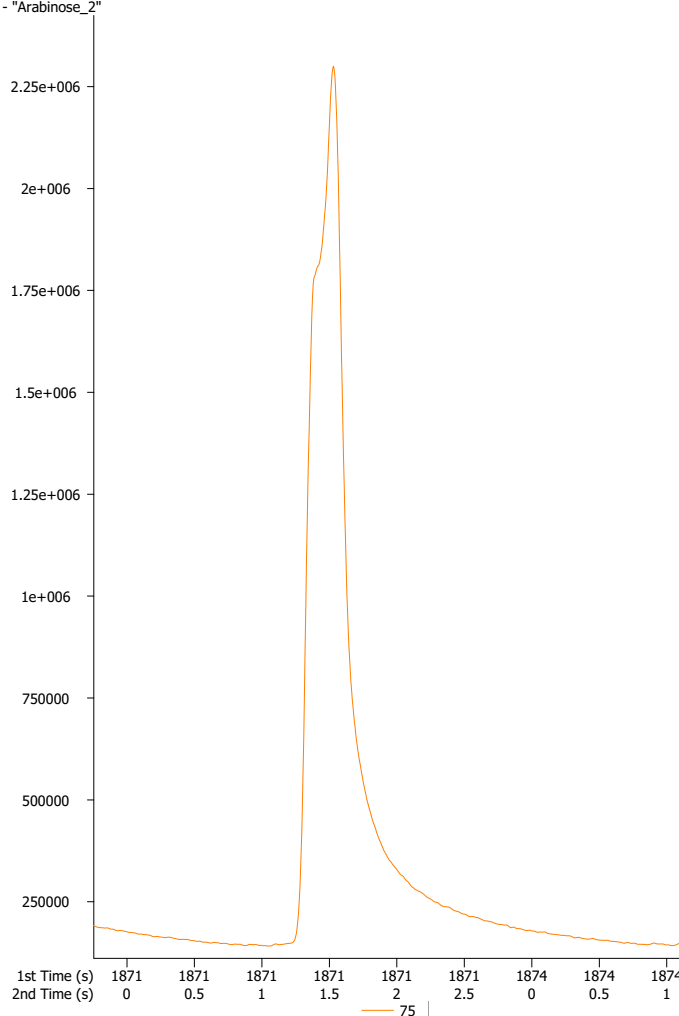

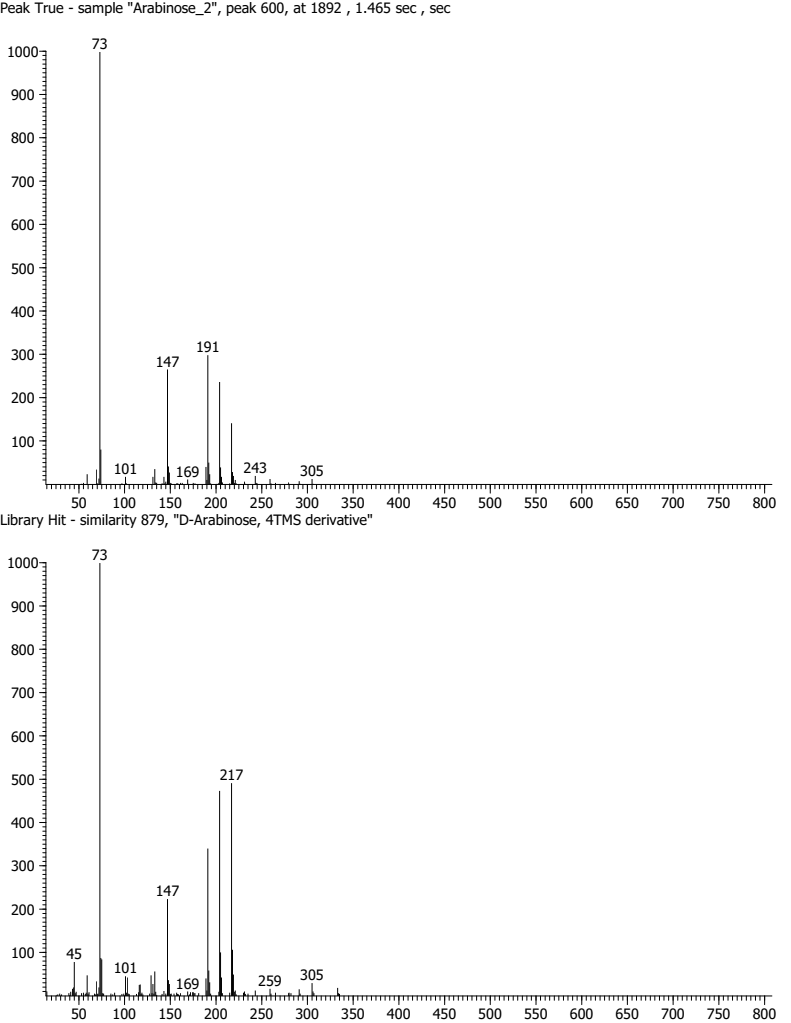


**Figure S2: Gas chromatogram (left) and MS pattern (right) of D-arabinose.**


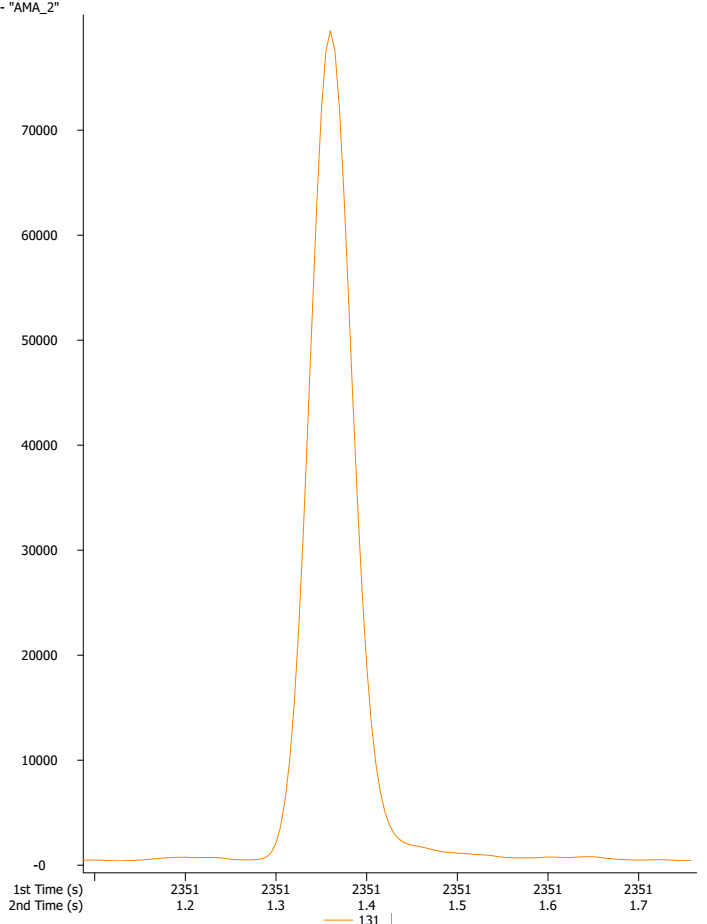

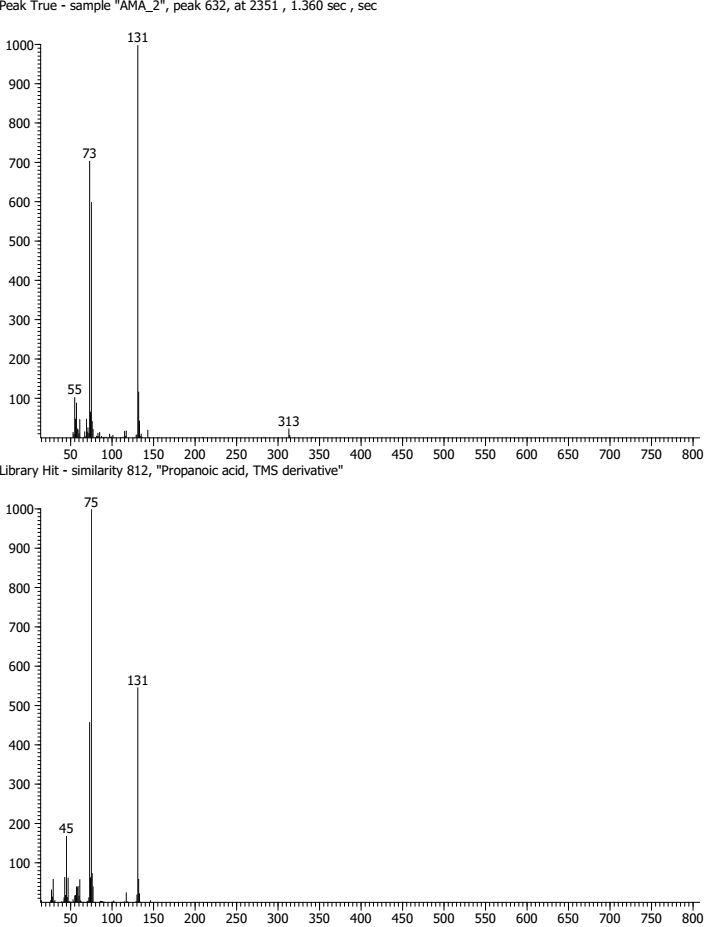


**Figure S3: Gas chromatogram (left) and MS pattern (right) of propanoic acid.**

**Figure S4: Gas chromatogram (left) and MS pattern (right) of propanoic acid.**


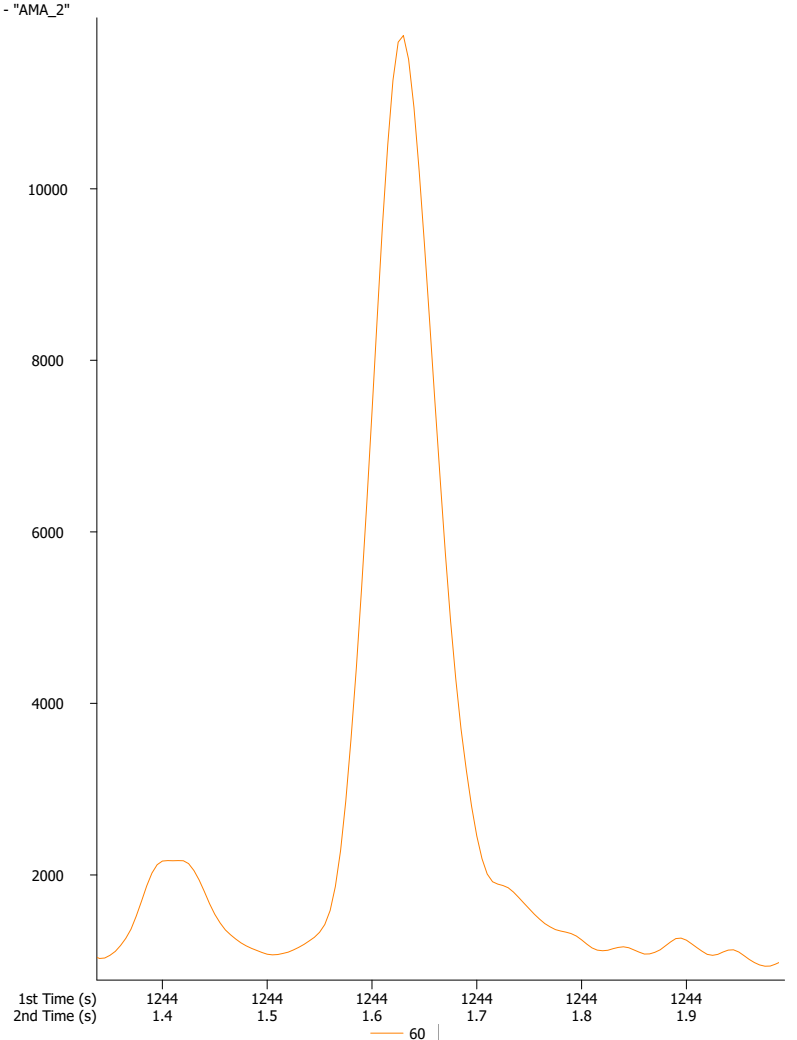

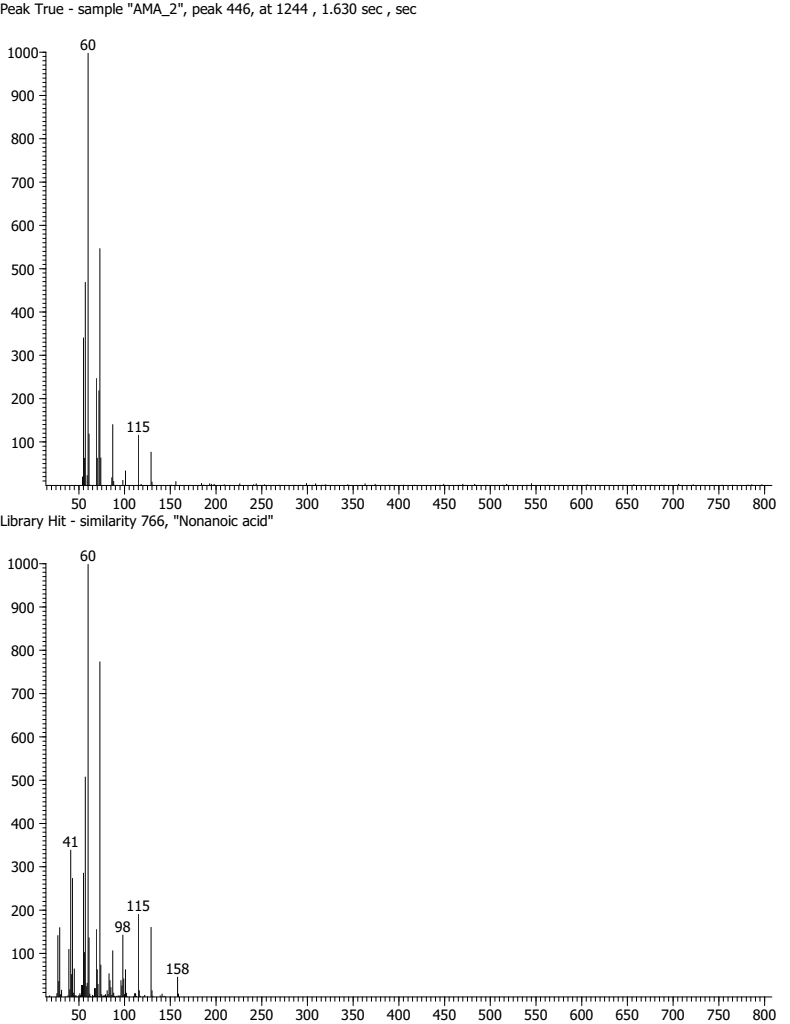

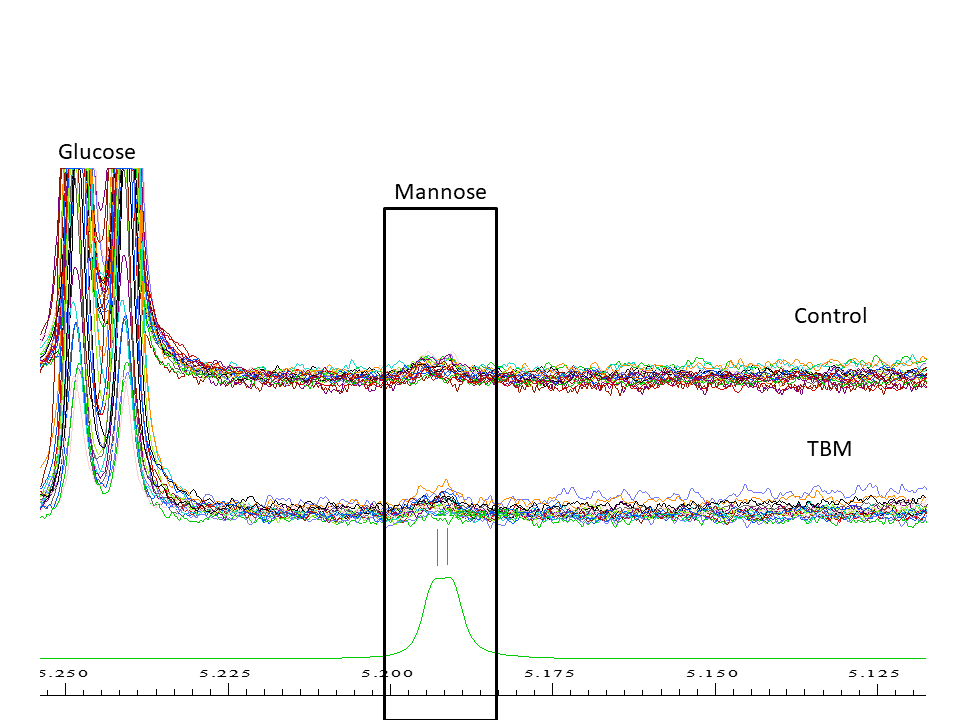

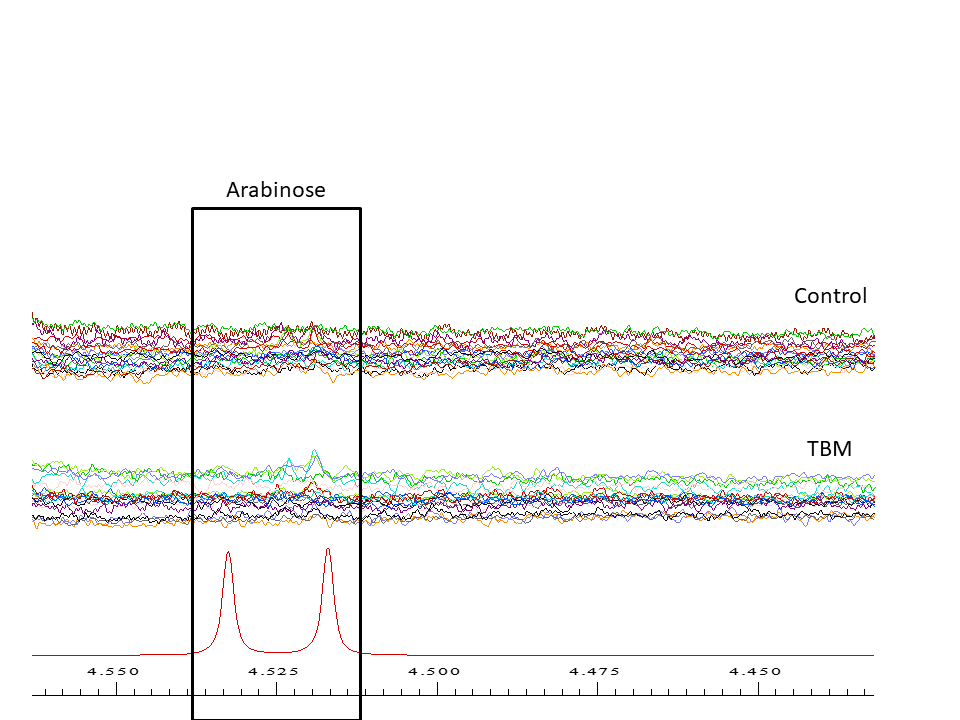


**Figure S5: ^1^H-NMR Spectra of D-Mannose (top) and D-Arabinose (bottom).**
